# Automatic Segmentation of Kidneys using Deep Learning for Total Kidney Volume Quantification in Autosomal Dominant Polycystic Kidney Disease

**DOI:** 10.1038/s41598-017-01779-0

**Published:** 2017-05-17

**Authors:** Kanishka Sharma, Christian Rupprecht, Anna Caroli, Maria Carolina Aparicio, Andrea Remuzzi, Maximilian Baust, Nassir Navab

**Affiliations:** 1 Department of Biomedical Engineering, IRCCS-Istituto di Ricerche Farmacologiche Mario Negri, Ranica (BG), 24020 Italy; 2 Computer Aided Medical Procedures, Technische Universität München, Garching bei München, 85748 Germany; 3 Department of Computer Science, Johns Hopkins University, Baltimore, 21218 USA; 4 Department of Management, Information and Production Engineering, University of Bergamo, Dalmine (BG), 24044 Italy; 5 Computer Aided Medical Procedures, Johns Hopkins University, Baltimore, 21218 USA

## Abstract

Autosomal Dominant Polycystic Kidney Disease (ADPKD) is the most common inherited disorder of the kidneys. It is characterized by enlargement of the kidneys caused by progressive development of renal cysts, and thus assessment of total kidney volume (TKV) is crucial for studying disease progression in ADPKD. However, automatic segmentation of polycystic kidneys is a challenging task due to severe alteration in the morphology caused by non-uniform cyst formation and presence of adjacent liver cysts. In this study, an automated segmentation method based on deep learning has been proposed for TKV computation on computed tomography (CT) dataset of ADPKD patients exhibiting mild to moderate or severe renal insufficiency. The proposed method has been trained (n = 165) and tested (n = 79) on a wide range of TKV (321.2–14,670.7 mL) achieving an overall mean Dice Similarity Coefficient of 0.86 ± 0.07 (*mean* ± *SD*) between automated and manual segmentations from clinical experts and a mean correlation coefficient (*ρ*) of 0.98 (p < 0.001) for segmented kidney volume measurements in the entire test set. Our method facilitates fast and reproducible measurements of kidney volumes in agreement with manual segmentations from clinical experts.

## Introduction

**Figure 1:**
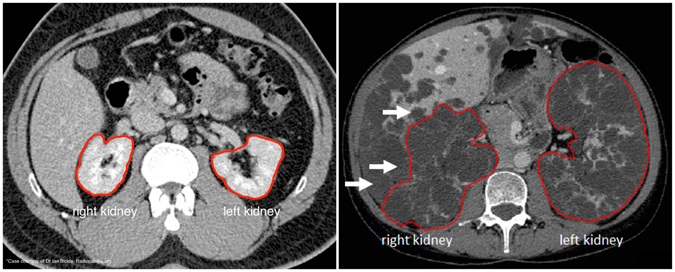
ADPKD Kidney Segmentation. ADPKD Kidneys (right panel) are difficult to segment due to severe morphological changes in comparison to healthy kidneys (left panel). White arrows show adjacent liver cysts exhibiting similar intensity.

Autosomal dominant polycystic kidney disease (ADPKD) is a systemic genetic disorder characterized by progressive enlargement of the kidneys caused by sustained development and expansion of bilateral renal cysts^1^. It is one of the leading causes of end-stage renal disease (ESRD)^2^ prompting dialysis or kidney transplantation in majority of the patients and is associated with extra-renal manifestations such as presence of cysts in the liver^3^. Renal Ultrasonography (US) is commonly performed for presymptomatic screening and assessment of ADPKD, however, imaging modalities such as Computed Tomography (CT) and Magnetic Resonance Imaging (MRI) offer higher spatial resolution thus facilitating the detection of smaller cysts^4^. Previous investigations have shown an association between total kidney volume (TKV) and renal function^5, 6^ and several studies have provided evidence for the use of TKV as an important imaging biomarker for assessment of disease severity as well as for predicting disease progression in ADPKD^7–10^. The European Medicines Agency (EMA) and the Food & Drug Administration (FDA) now acknowledge TKV as prognostic imaging biomarker for use in clinical trials^11, 12^. In order to evaluate the efficacy of novel therapies, it is therefore crucial to develop rapid and reliable methods for TKV quantification. However, segmentation of polycystic kidneys for quantifying kidney volumes is very challenging due to non-uniform renal cyst growth leading to high variability in kidney morphology. Polycystic kidneys are characterized by their markedly irregular shape and size in comparison to normal kidneys and sometimes surface irregularities are prominent due to the presence of surface cysts of different size. On both CT and MRI, most frequent clinical complications for an automated assessment of TKV include the presence of hepatic cysts (Fig. 1) which appear identical to kidney cysts as well as the presence of hemorrhagic renal cysts which appear rather dissimilar to other fluid filled cysts leading to high intensity variability within the kidney. Thus, development of a fully-automated segmentation method for fast and precise TKV estimation remains a challenging problem.

In ADPKD studies, traditional methods for TKV computation based on CT and MRI acquisitions are stereology^13^ and manual segmentation. For stereology, a square grid with user-specified cell positions and cell spacing is superimposed on every slice and the TKV is estimated by manually counting all grid points covering the kidney region. The accuracy of this method depends on user-specified parameters such as the cell spacing. Manual segmentation requires delineating the kidney on every slice using either an available free-hand contouring tool or an interactive segmentation method that guides the operator while delineating the region of interest. Both stereology and manual segmentation are tedious and require expert training in order to achieve an accurate assessment of TKV. As a consequence, several methods have been proposed with the goal of providing sufficiently accurate but less tedious means for TKV computation. The mid-slice method^14^ and the ellipsoid volume equations^15, 16^ have been proposed for estimating TKV from 1–2 slices. Even though these methods facilitate fast quantification, they provide only a rough estimate of TKV which could be convenient for clinical management but inadequate for clinical trials to detect smaller changes estimated to be approximately 5%^7, 10^ per year. Besides these approaches, segmentation of polycystic kidneys using semi-automated methods on MRI and CT have been reported previously. Among semi-automatic approaches on MRI, Daum *et al*.^17^ used 3D random walks while Racimora *et al*.^18^ proposed active contours and morphological operations for segmentation of polycystic kidneys. Moreover, methods based on region-growing and curvature motion have previously been suggested^19, 20^. Recently, geodesic active contours coupled with watershed edge detection have been used for semi-automatic segmentation on T2 weighted MR images^21^. On CT, Sharma *et al*.^22^ reported an approach based on random forests and geodesic distance volumes for 3D segmentation of polycystic kidneys from ADPKD patients with severe renal insufficiency. Among automatic approaches, Kline *et al*.^23^ described segmentation of follow-up MR images using initialization from previously performed manual segmentations of baseline MR acquisitions. In another recent work, Kim *et al*.^24^ reported a level set framework for automatic segmentation of polycystic kidneys on relatively small MRI dataset of ADPKD patients. Majority of these segmentation approaches focus on MRI and to the best of our knowledge, no study in ADPKD has evaluated a fully automated segmentation method both quantitatively and qualitatively on CT.

In recent years, Convolutional Neural Networks (CNNs) have shown superior performance in several computer vision tasks such as image classification, object detection and semantic segmentation. The main advantage of CNNs in comparison to many other machine-learning-based methods, such as random forests, is that they do not require hand-crafted features. Previous works have been reported on pixelwise labelling of objects for semantic segmentation using CNN^25–27^. In the domain of medical imaging, CNNs have previously been proposed for localization and segmentation of kidneys with mild morphological changes using patch-wise approaches on CT^28, 29^.

In this work, we present automated segmentation of ADPKD kidneys using fully convolutional neural networks, trained end-to-end, on slicewise axial-CT sections. We report the performance of our method quantitatively and qualitatively on 244 CT acquisitions of patients exhibiting different stages of ADPKD.

## Results

For our experiments, baseline and follow-up CT acquisitions (training set = 165; test set = 79) of ADPKD patients (n = 125) with a wide range of TKV (321.2 mL–14,670.7 mL) and an estimated glomerular filtration rate (eGFR) ≥ 40 *ml*/*min* per 1.73 *m* ^2^ (Study 1) or 15 ≤ *eGFR* ≤ 40 *ml*/*min* per 1.73 *m* ^2^ (Study 2 and Study 3) were used. The main demographic and clinical characteristics of the patients are summarized in Table 1

**Table 1:** Demographics and Clinical Characteristics of ADPKD Patients.

Clinical Study	Gender Female/Male	Number of Acquisitions Training Set Test Set	Age (years) Mean Age [Range]	Estimated Glomerular Filtration Rate (eGFR) (ml/min per 1.73 m^2^)	Total Kidney Volume (mL) Mean ± SD [Range]
**SIRENA** (study 1)	3/12	26 26	39.1 [28–46]	eGFR ≥ 40	1,891.4 ± 1,073.2 [501.9–5,093.2]
**SIRENA 2** (study 2)	24/17	45 15	53.8 [41–70]	15 ≤ eGFR ≤ 40	3,139.1 ± 1,485.5 [1,197.1–6,634.1]
**ALADIN 2** (study 3)	32/37	94 38	53.6 [33–74]	15 ≤ eGFR ≤ 40	3,132.7 ± 2,152.2 [321.2–14,670.7]

ADPKD patients (n = 125) with baseline and follow-up CT acquisitions (training set = 165, test set = 79) included in our study.

. The results of study 2 and study 3 were combined together as patients in both studies show similar clinical characteristics.

### Segmentation Similarity Analysis

**Figure 2:**
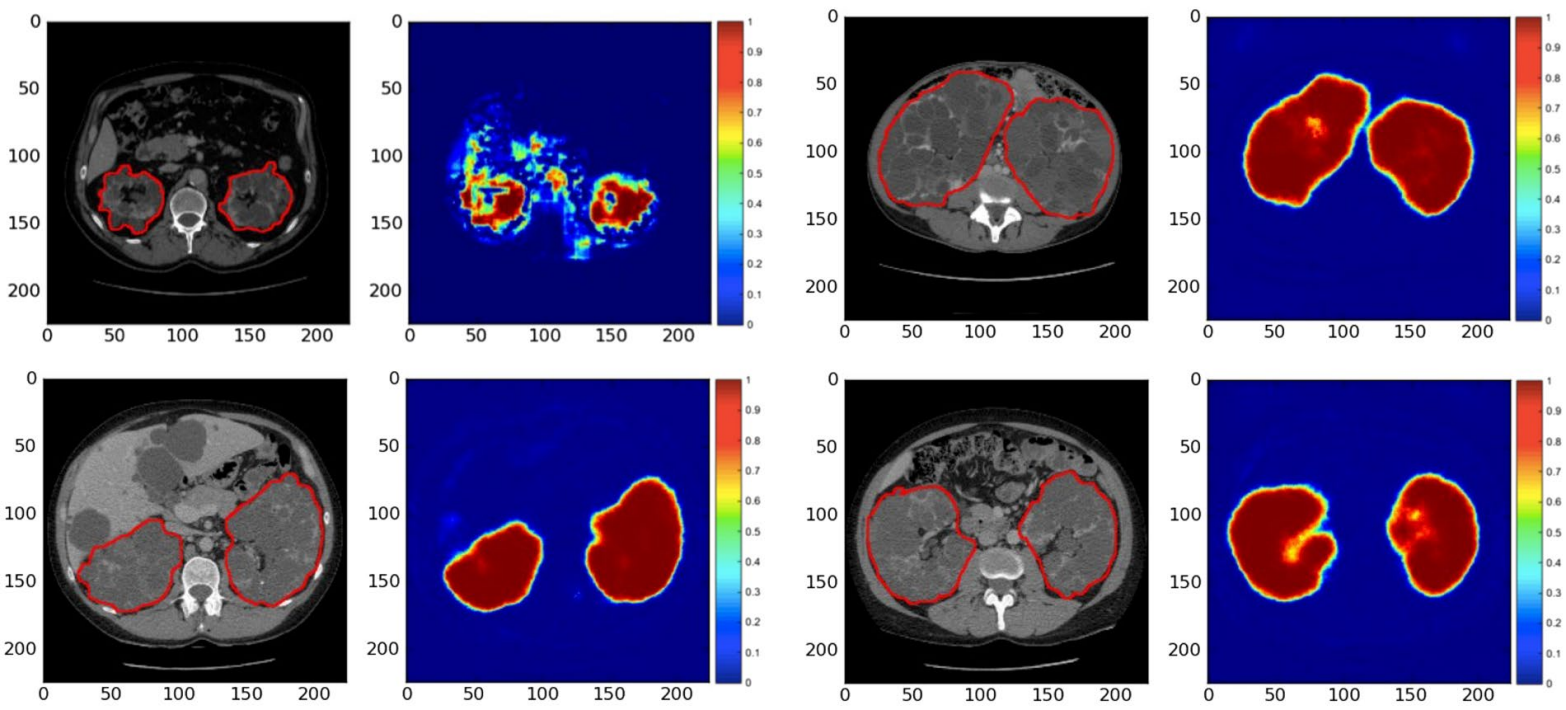
CNN predictions of ADPKD Kidneys. Four segmentations (red contour) of ADPKD kidneys from CT acquisitions of different patients are shown. The corresponding CNN-generated probability maps are shown in pseudo colors.

In Fig. 2, segmentation predictions from the CNN of 4 different patients have been shown. The time required for segmentation prediction using automated segmentation method was only few seconds per patient CT acquisition, while the manual segmentation from experts required approximately 30 minutes per patient. The overall mean Dice Similarity Coefficient (DSC) between segmentations from the automated method and ground truth kidney segmentations from clinical experts was 0.86 ± 0.07 (mean ± SD) for the entire test set (n = 79). In particular, for study 1, consisting of patients with mild to moderate renal insufficiency (n = 26, Table 1), the mean DSC was 0.86 ± 0.06. Combining study 2 and study 3, consisting of patients with moderate/severe renal insufficiency (n = 53, Table 1), the mean DSC was 0.86 ± 0.08.

### TKV Agreement Analysis

**Figure 3:**
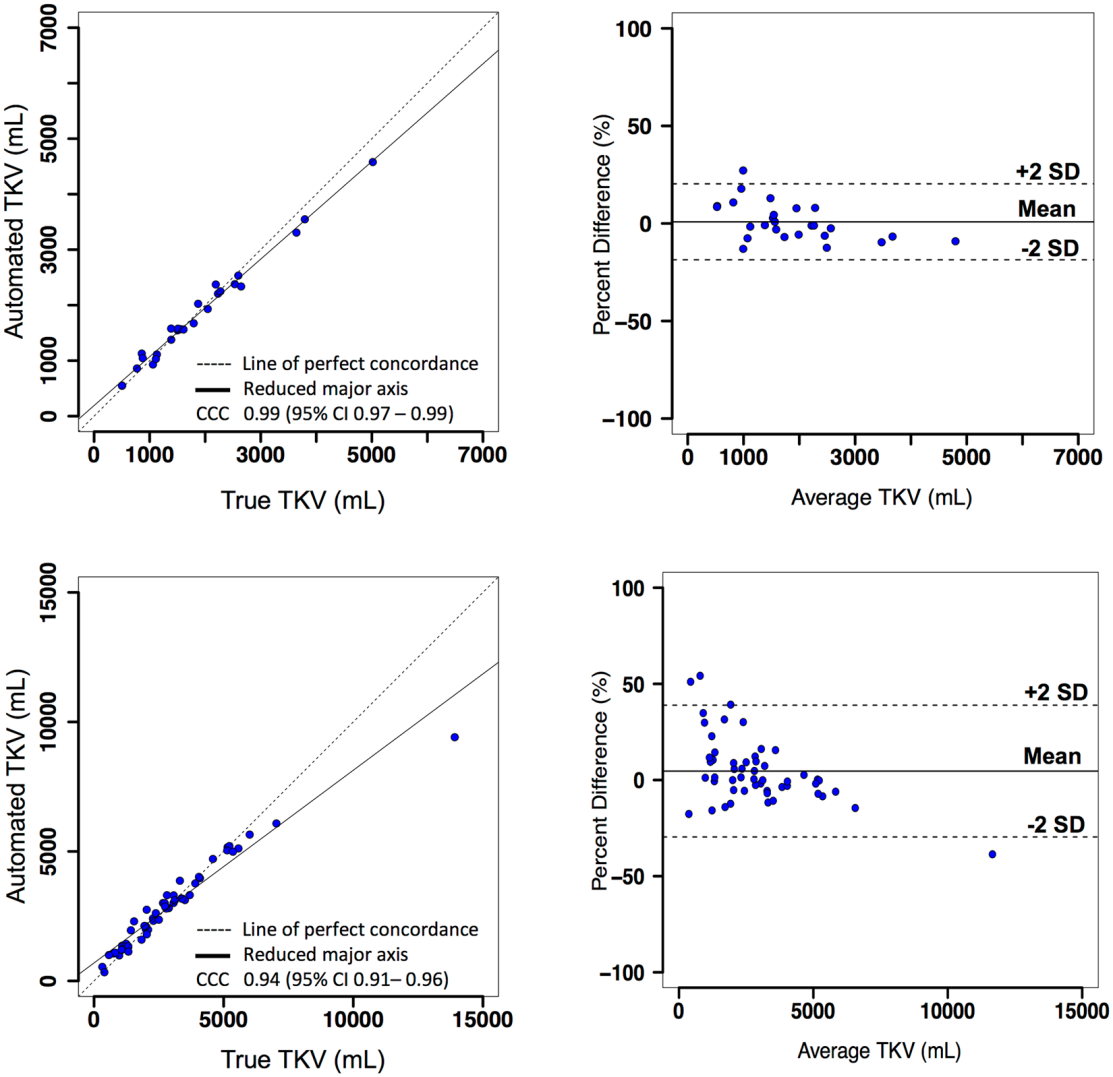
Left: Concordance Correlation Coefficient (CCC) plots showing strength of association; Right: Bland-Altman plots showing agreement between TKV measurements. TKV measurements from automated segmentation method (main experiment) are compared with true TKV measurements from manual segmentations for study 1 (top, n = 26) and, studies 2 and 3 (bottom, n = 53).

We performed volumetric measurement on kidney segmentations from the CNN and compared the automated TKV with the true TKV (obtained from ground truth annotations) in terms of accuracy and precision of the measurement. For study 1, as shown in Fig. 3 (top-left), there is substantial strength of association between the automated and true TKV with a concordance correlation coefficient (CCC) of 0.99 [95% Confidence Interval (CI): 0.97–0.99]. For the test set (n = 26, Table 1), the mean TKV error between automated and true measurements was −32.9 ± 170.8 mL and the mean percentage TKV error was 1.3% ± 10.3%. In addition, the mean absolute percentage error (MAPE) was 7.8% ± 6.7%. Bland Altman plots were used to further determine the agreement between the two methods. For study 1, as shown in Fig. 3 (top-right), the lower and upper limits of agreement (LOA) for percentage difference were −18.6% and 20.3%, respectively. The coefficient of variation (COV) between true and automated TKV was 6.5%.

For studies 2 and 3, in Fig. 3 (bottom-left), the test cases (n = 53, Table 1 with studies 2 and 3 combined) from automated and true TKV measurements show moderate strength of association with a CCC of 0.94 [95% CI: 0.91–0.96]. The mean TKV error between automated and true measurements was −44.1 ± 694.5 mL while the mean percentage TKV error was 6.5% ± 20.1%. The overall MAPE was 13.3% ± 16.4%. On the Bland-Altman plot, Fig. 3 (bottom-right), the lower and upper LOA were −29.6% and 38.9%. The overall COV between true and automated TKV was 17%.

**Figure 4:**
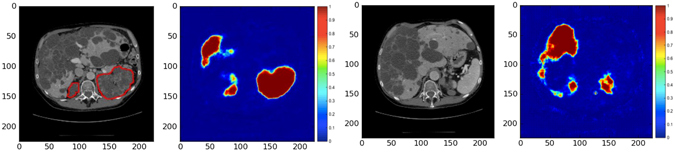
Mislabelled predictions by CNN. Left: Liver cysts predicted as foreground along with kidney region; Right: Cystic liver mislabelled as Kidney.

The difference in the TKV measurements was found to be statistically insignificant for all the three clinical studies (p > 0.05). The automated TKV measurements showed high positive correlation with true TKV measurements for study 1 with mean correlation coefficient (Spearman’s rho) *ρ* = 0.97 (p < 0.001). Similarly, high positive correlations were observed for studies 2 and 3, with *ρ* = 0.98 (p < 0.001). In some cases with CT acquisitions containing severe liver cysts in close proximity of the kidney, the kidney volume was over-estimated due to inclusion of these cysts as false positive regions in the kidney segmentation. Example predictions mislabeled by the CNN have been shown in Fig. 4.

### Cross Validation Analysis

In order to confirm the performance from the main experiment, we performed an additional 3-fold cross-validation. The details and results of the cross-validation analysis have been summarized in Table 2

**Table 2:** Cross Validation Experiments.

	Main Experiment	Cross Validation 1	Cross Validation 2	Cross Validation 3
**TRAINING**				
Total Acquisitions	165	162	161	161
Total Original images	16,000	16,079	15,706	15,544
Training images: Including Augmentation	48,000	48,237	47,118	46,632
**TESTING**				
Total Acquisitions	79	80	81	81
Total Original images	9,000	8,634	9,007	9,169
**Mean TKV (mL)**				
(mean ± SD)	2,549.0 ± 1,951.1	2,729.0 ± 1,499.3	2,780.0 ± 1,560.0	2,816.4 ± 1,669.7
[Range]	[321.2–13,913.6]	[399.0–7,034.5]	[501.9–7,480.6]	[321.0–9,605.6]
**Dice Score Coefficient (DSC)**				
(mean ± SD)	0.86 ± 0.1	0.86 ± 0.1	0.83 ± 0.8	0.87 ± 0.6
**Mean Percentage Error (MPE)**				
(mean ± SD)	4.8 ± 17.6	0.2 ± 21.1	6.8 ± 20.8	−1.3 ± 18.9
**Mean Absolute Percentage Error (MAPE)**				
(mean ± SD)	11.5 ± 14.1	15.1 ± 14.5	15.0 ± 15.8	13.5 ± 13.1
**Concordance Correlation Coefficient (CCC)**	0.95	0.93	0.91	0.94
**Coefficient of Variation (COV)**	16	14	15	15
**Root Mean Square Error (RMSE)**	573	527	587	564
**Root Mean Square Percentage Error (RMSPE)**	18	21	22	19

3-fold cross-validation to assess the performance of our fully convolutional neural network.

. The DSC for cross-validation sets were 0.86 ± 0.1, 0.83 ± 0.8 and 0.87 ± 0.6, respectively. The MAPE ranged from 13.5 to 15. The COV for all three sets ranged from 14 to 15 while the root mean squared percentage error (RMSPE) ranged from 19 to 21.

## Discussion

In this study, we present a novel method to automatically segment kidneys, and investigate its qualitative and quantitative accuracy and precision to measure TKV on a large dataset of CT acquisitions from ADPKD patients. The annual increase in TKV has been estimated to be around 5%^7, 10^ per year, suggesting that TKV measurement should be accurate to capture small changes over time. The most commonly used methods for kidney volume computation such as manual delineation and stereology^13^ are simple but time consuming and subject to intra/inter-observer variability. Alternatively, the mid-slice method^14^ and the ellipsoid equation^15, 16^ serve to provide quick TKV measurement but lead to low accuracy and precision compared to whole kidney segmentation.

On MRI, Racimora *et al*.^18^ proposed a segmentation approach yielding mean percentage TKV error of 22.0% ± 8.6% with an automated active contour algorithm that reduced to 3.2% ± 0.8% after manual post-editing efforts. Another semi-automatic approach with geodesic active contours and watershed edge detection^21^ achieved high accuracy with mean TKV difference of 0.19% ± 6.96%. Mignani *et al*.^19^ compared their results with stereology and reported mean percentage TKV error of −0.6 ± 5.8%, while, Turco *et al*.^20^ reported MAPE of 4.4% ± 4.1% and 4.2% ± 4.0% for the left and right kidneys, respectively. Other supervised segmentation methods based on stereology^30^ on MRI and random forests on CT^22^ have also been reported previously. However, these semi-automatic techniques are subject to intra/inter-observer variability and mostly require post-processing efforts to achieve higher accuracy leading to increase in overall processing time of TKV. Kline *et al*.^23^ proposed automatic segmentation on follow-up MR images, however, their method essentially requires previously performed manual segmentations of kidneys on baseline images as initialization for the segmentation process. In the work of Kim *et al*.^24^, a level set framework has been proposed for automatic segmentation in ADPKD. Even though their method shows good correlation between automated and manual TKV measurements, the results indicate high variability (LOA higher than ±25%) when compared with the manual method. Zheng *et al*.^29^ used patch-based CNN in combination with marginal space learning for localization of pathological kidneys prior to an active shape model for segmentation. Their results show good segmentation accuracy (DSC > 0.88) but there is substantial increase in segmentation error without CNN initialisation. Also, the presented dataset in their work appears to contain kidneys with milder morphological changes.

In this study, we trained and assessed the performance of the proposed automated segmentation method both quantitatively and qualitatively on a large CT dataset (n = 244) of patients at different stages of ADPKD, using manual segmentations from clinical experts as gold standard. In comparison with majority of the methods previously reported on TKV computation in ADPKD, our method has been evaluated on a larger TKV range (>300 mL and <15,000 mL). For study 1 with ADPKD patients at early stage of the disease and TKV range between 500 mL and 6,000 mL, the automated TKV shows very high strength of association (CCC = 0.99) with true TKV, however, for studies 2 and 3, with ADPKD patients at more advanced stage of the disease and the TKV range between 300 mL and 15,000 mL, there is moderate strength of association (CCC = 0.94) between the two methods. Similarly, the overall accuracy and precision of the TKV measurements from automated method is higher for study 1 (MAPE = 7.8% ± 6.7%; COV = 6.5%), compared to studies 2 and 3 (MAPE = 13.3% ± 16.4%; COV = 17.0%). The performance of the automated method is decreased particularly for very low TKV (<500 mL) and for extremely high TKV (>10,000 mL). This can be attributed to availability of very few instances of such small or very huge kidneys leading to poor predictions by the CNN during testing phase. However, the overall difference in TKV measurements was found to be statistically insignificant (p > 0.05) for all three clinical studies and the automated TKV measurements show high positive correlation with true TKV measurements (*ρ* = 0.98, p < 0.001). Moreover, the proposed method takes only few seconds for prediction of segmentation on each patient acquisition and avoids any intra/inter operator segmentation bias.

Despite the promising results, our study has some limitations. In some cases with several liver cysts in close proximity of the kidney (Fig. 4), the automated segmentation method over-estimated the kidney volume due to inclusion of liver cysts in the segmented kidney region. To potentially overcome this problem, the proposed method can be trained on 3D volumes of polycystic kidneys.

**Figure 5:**
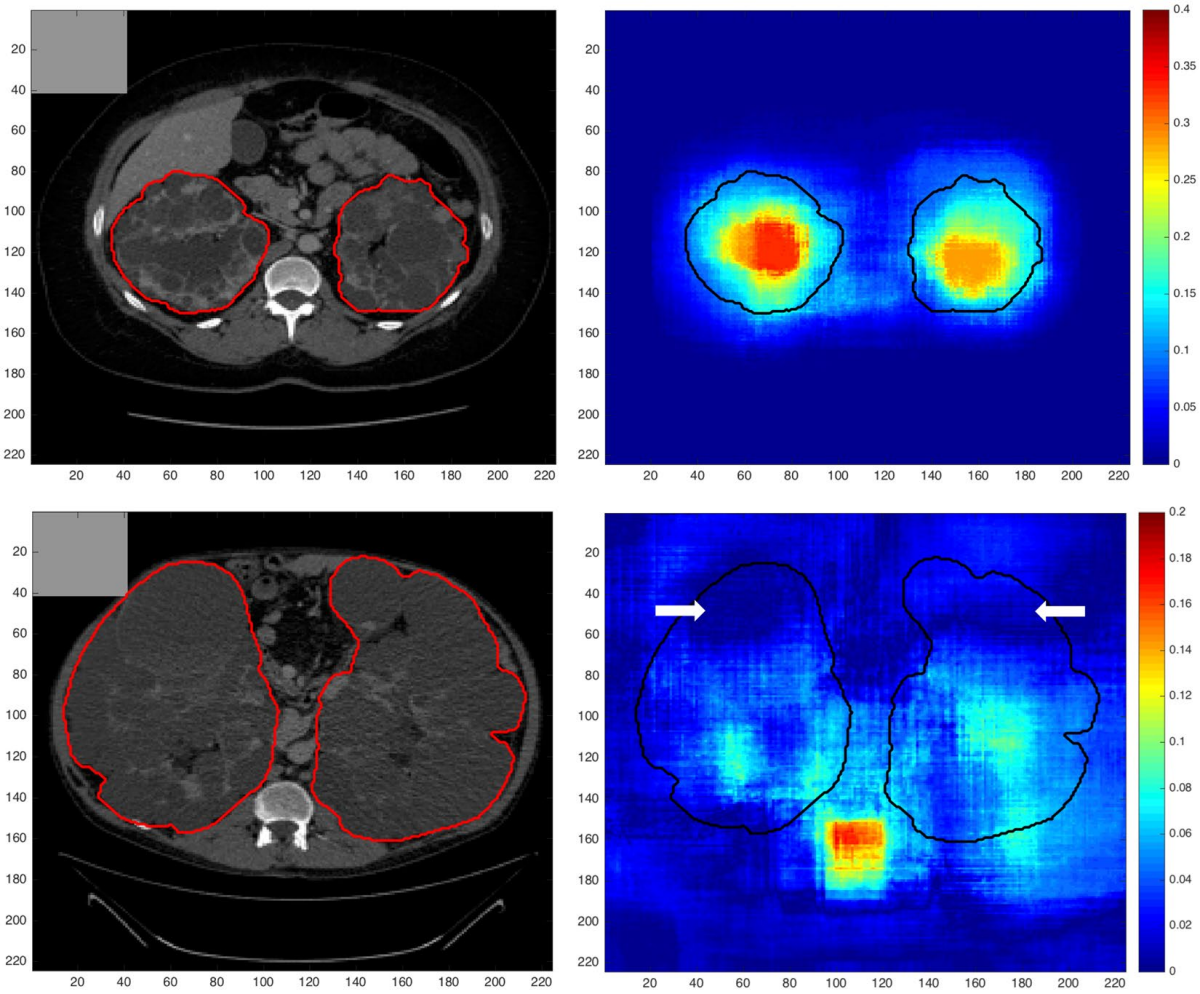
Feature Visualization. Segmentation maps measuring change in DSC while occluding (shown with gray square) different parts of the image with respect to original unoccluded image as a measure of importance for the respective image region. The manually generated outline of the kidney is shown in red and black, respectively. Top: The largest change occurs when the kidneys themselves are occluded. Bottom: Same experiment for an ADPKD patient with high TKV (>13,000 mL).

Regarding the importance visualization in Fig. 5, we see the importance of context for the segmentation especially for very rare subjects with extremely high TKV: In case of a typical patient, we see in Fig. 5 (top) that the largest change occurs when the kidneys themselves are occluded. This is not only intuitive but also confirms that the network is not confused by changes far away from the regions of interest. This highlights robustness against changes far away from the region of interest. Nonetheless, the visualized influence region extends over the object boundaries which indicates that not only the kidneys themselves, but also local context is used to find the final segmentation. For very rare cases with extremely high TKV (>13,000 mL) though, the spatial context changes entirely due to both kidneys occupying most of the abdominal region. As a consequence, the CNN cannot exploit context information leading to poor segmentation results which is also confirmed by the feature visualization experiment (Fig. 5 (bottom)): Consider particularly the upper areas (indicated by white arrows) in the annotated kidneys which exhibit low variation as kidney tissue does typically not appear in these areas indicating that the CNN is not expecting kidney tissues in this area.

Another limitation of our study is that we performed the analysis only on CT. Future work is needed to extend the proposed method to MRI, by training the CNN and specifically tune the parameters used during training for MRI images. Finally, despite using CT images with varying image quality to train the algorithm, the segmentation method turned out to be sensitive to image quality. In conclusion, we presented a fully automated method for the segmentation of polycystic kidneys from patients at different stages and severity of ADPKD using CT data. Our method can be reliably used on a TKV range of >500 and <10,000 mL, facilitating fast and reproducible measurements of kidney volumes in agreement with manual segmentations from clinical experts. The overall segmentation can be further improved by incorporating user interaction to correct mislabelled sections of CT. Particularly for high resolution CT images, this can significantly reduce the TKV computation time compared to manually tracing every section of the kidney and also, capture smaller changes in TKV over time. As a future work, the automated method can be trained on other affected organs such as the polycystic liver for computation of the liver volume in ADPKD.

## Methods

### Patients and CT Image Acquisition

The ADPKD patient dataset (244 CT acquisitions) used for our experiments was obtained from three different ADPKD clinical studies (Table 1): study 1 (SIRENA), study 2 (SIRENA 2), and study 3 (ALADIN 2).

The SIRENA study^31^ (ClinicalTrials.gov Identifier: NCT00491517) was designed to assess the risk/benefit of mTOR inhibitor therapy on disease progression in ADPKD. This randomized, crossover study compared 6-month treatment with sirolimus or conventional therapy alone on the increase in kidney volume and its compartments in adult patients with ADPKD and normal renal function or mild to moderate renal insufficiency (*eGFR* ≥ 40 *ml*/*min* per 1.73 *m* ^2^). The SIRENA 2 study^32^ (ClinicalTrials.gov Identifier: NCT01223755) was an academic, prospective, randomized, open label, blinded end point, parallel group trial that recruited adults with ADPKD and CKD stage 3b or 4. The aim of the study was to compare changes in GFR on 3-year treatment with sirolimus added on to conventional therapy or conventional treatment alone in ADPKD patients with moderate/severe renal insufficiency(15 ≤ *eGFR* ≤ 40 *ml*/*min* per 1.73 *m* ^2^). The ALADIN 2 Study (ClinicalTrials.gov Identifier: NCT01377246) is an ongoing multicenter, randomized, long-term (3-years) longitudinal study that recruited adult ADPKD patients with moderate/severe renal insufficiency (15 ≤ *eGFR* ≤ 40 *ml*/*min* per 1.73 *m* ^2^). The aim of this study is to assess the efficacy of 1 year treatment with long-acting somatostatin analogue (Octreotide LAR) compared with placebo in slowing kidney and liver growth rate in the ADPKD patients and to assess whether and to which extent this translates into slower renal function decline over 3-year follow-up. The CT images in all these clinical studies were acquired with a 64-slice CT scanner (LightSpeed VCT; GE Healthcare, Milwaukee, WI). A single breath-hold scan (120 kV; 150 to 500 mAs; matrix 512 × 512; collimation 2.5 mm; slice pitch 0.984; increment 2.5 mm) was initiated 80 seconds after the injection of 100 ml non-ionic iodinated contrast agent (Iomeron 350; Bracco, Italy) at a rate of 2 ml/s, followed by 20 ml physiologic solution at the same injection rate. The CT images from study 1 and study 2 were acquired at single centre in Bergamo, while CT images from study 3 were acquired at four different centres (Bergamo, Naples, Agrigento and Treviso) in Italy.

### Data Annotation and Patient Selection

The left and the right kidneys were outlined manually by clinical experts and trained personnel using the ImageJ software (version 1.48v)^33^. In order to segment the kidneys, the image sequence for each CT acquisition was accessed using ImageJ software and manually delineated along the kidney border. The boundary delineation was performed using a standard protocol for all kidneys with respect to the hilum and liver cysts. To avoid inter-rater variability in the dataset, all manual segmentations were finally checked and corrected by a single operator (KS).

For our main experiment, patients were manually divided into the training and test set, trying to achieve a similar distribution in both sets based on the available TKV range (321.2 mL–14,670.7 mL), see Table 2.

### Convolutional Neural Network Architecture

**Figure 6:**
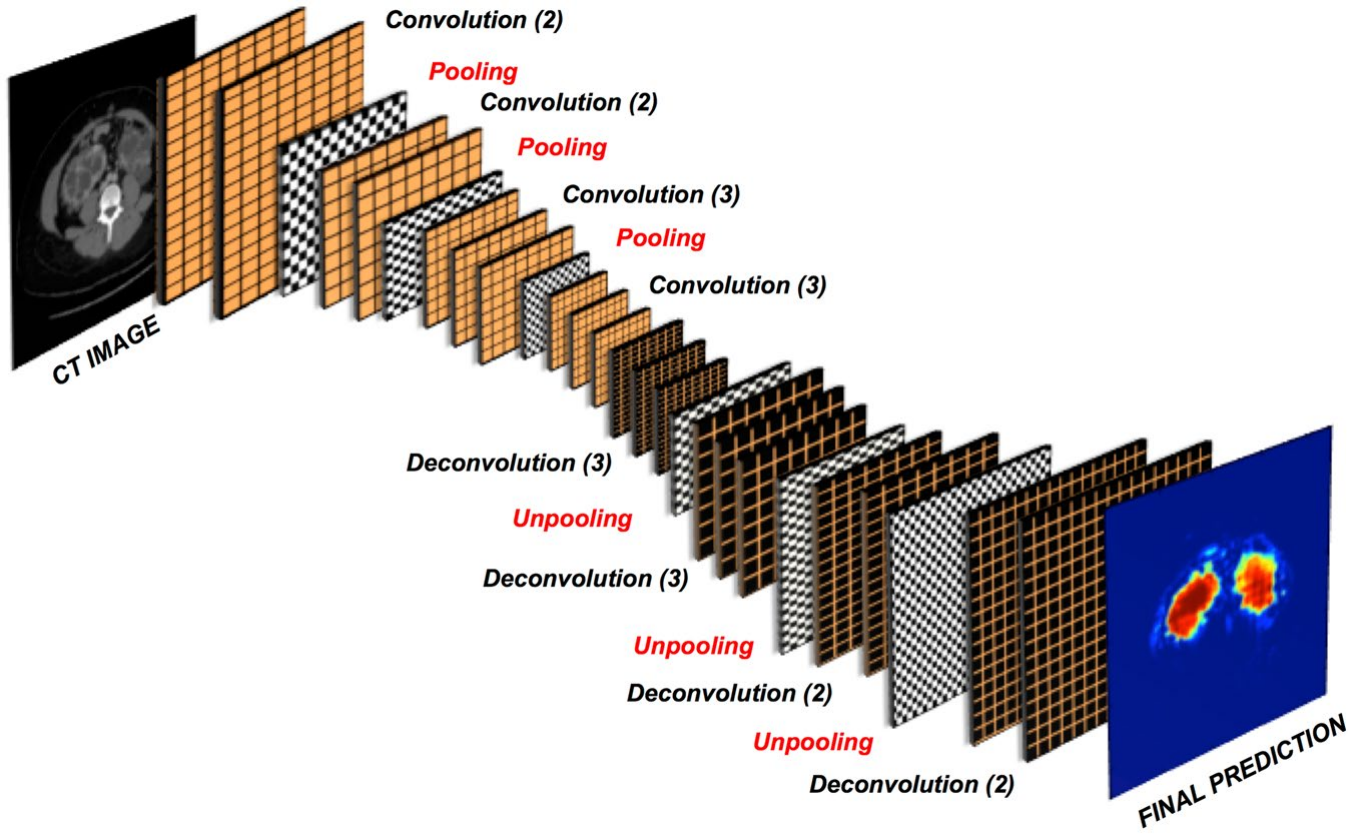
Fully Convolutional Neural Network Architecture. For feature extraction step, we used 10 layers of convolution filters with a receptive field of 3 × 3 and spatial padding of 1 pixel followed by max pooling layers with 2 × 2 pixel window and stride of 2 pixels to progressively reduce the spatial size of the input after convolution step. To achieve pixelwise segmentation, deconvolution and unpooling layers were used for upsampling the feature maps.

CNNs consist of several layers that learn hierarchy of features without relying on handcrafted features. Taking images as input, they convert raw pixels to final class scores by passing them through series of learnable convolutional filters. In order to perform tasks such as segmentation, it is necessary to obtain pixel-wise classification from the CNN. Our CNN architecture, shown in Fig. 6, follows the VGG-16 representation^34^ but employs only the first 10 layers comprising of convolution filters with a small receptive field of 3 × 3 and a spatial padding of 1 pixel for every convolution layer. As explained in the work of Ioffe *et al*.^35^, due to the internal covariance shift problem, it is rather difficult to train deep neural networks with saturating non-linearities. To alleviate this problem, batch normalization after every convolution layer allows proper initialization of the network by the forcing the activations to a standard Gaussian distribution and normalizing the inputs to zero mean and unit variance. In our experiments, we observed that application of batch normalization was crucial to improve the overall accuracy of the network. Each batch normalization layer is followed by a layer of neurons with Rectified Linear Unit (ReLU)^36^ as the activation function. In order to reduce the number of parameters, and thus the computation complexity of the network, max-pooling layers with a 2 × 2 pixel window and a stride of 2 pixels were used to progressively reduce the spatial size of the input to half the original size along both the height and the width. The described layers correspond to the feature extraction step, and to achieve pixelwise segmentation we employed series of deconvolution and unpooling layers for up-sampling the feature maps following the work of Zeiler *et al*.^37^ and Noh *et al*.^38^. The deconvolution layers essentially perform convolution like operation but in the opposite way leading to upsampling of coarse feature map into the reconstructed shape of the input. However, performing deconvolution step alone leads to a rather coarse reconstruction and therefore, the unpooling layers are required to refine the output. These unpooling layers reverse the operation of the pooling layers by preserving the locations of maximum activation that were extracted during the max-pooling step, and re-utilise these locations to place the maximum activations back to their original spatial position^37^. The unpooling layers and the deconvolution layers are connected to a 1 × 1 convolutional layer that maps the final feature vector to the desired foreground (kidney) and background (non-kidney) classes. The output of this final convolutional layer is connected to a multinomial logistic loss/cross-entropy loss to optimize the weights by penalizing the deviation between true and predicted labels.

### Data Augmentation, Training and Testing

In order to mitigate overfitting and to achieve a good generalisation, we performed two different augmentation methods on the training dataset; firstly by image shift in x-y direction and secondly by non-rigidly deforming the respective slice and applying a low frequent intensity variation. This increases the training dataset from 16000 CT slices to 48000 CT slices in case of main experiment. All this data is used for network training and allows it to learn desired invariances such as shift invariance or variable polycystic kidney shapes. Detailed information about the augmentation procedure is available as Supplementary information. All experiments were performed using the Caffe^39^ framework. In order to reduce the computational complexity of the network, the original size of the CT slices 512 × 512 was re-sampled to 224 × 224 and the image range was normalized to [0,255]. Additionally, we performed mean subtraction from the training dataset as a pre-processing step. Furthermore, slices were randomly shuffled before feeding to the CNN. Using a sampling size of 8 (batch-size) for each iteration, training was performed on a workstation with an Intel Xeon 8-core 2.40 GHz CPU and a NVIDIA GeForce TITANX (12 GB) GPU, taking approximately 24 hours to train the network. For optimization, we used the adaptive gradient (“AdaGrad”) method, with the learning rate set to 0.0001, and a weight decay of 0.0005, respectively. We initialized the weights in the convolution and the deconvolution layers using the Xavier initialization^40^.

For the test phase, the previously unseen 9000 CT slices (79 CT acquisitions) that were not included in the original training phase were passed through the trained network. The output prediction consisted of the foreground (kidney) and the background (non-kidney) pixels, where pixels with a probability higher than 0.5 were regarded as foreground (kidney) pixels. The threshold was selected based on the analysis of the Receiver Operating Characteristic (ROC) space. In conjunction to the ROC space, we also computed the Accuracy, Precision, F1 Score and the Youden-Index. Details about the threshold selection have been included in the Supplementary information.

### Cross Validation

We performed an additional 3-fold cross validation on the dataset (Table 2). Out of the 244 acquisitions, 2 cases (TKV > 13,000 mL) were removed from the dataset as they are not representative over the patient population and thus do not provide adequate number of images for learning these rare cases. This was confirmed by a feature visualization experiment for one of these patients as shown in Fig. 5 (bottom). In order to perform the cross validation, the dataset was sorted according to ascending TKV range and then randomly partitioned into 3 subsets (n = 242: 80, 81, 81) allowing splitting of the dataset uniformly into the 3 cross-validation sets. For each experiment, 2 sets were used for training and the remaining set for testing. This process was repeated three times such that every data set was used once for testing.

### Feature Visualization

To gain insight into the learned features and particularly the importance of context, we adapted the occlusion method from Zeiler and Fergus^37^ to the image segmentation domain. Their approach measures the change in classification while occluding the image with a square of fixed size and constant intensity value in a sliding window manner in order to derive the importance for each image region. Hereby in Fig. 5, we retrieve full segmentation maps from the network instead of classification scores. Thus, we compute the change in DSC with respect to the original, unoccluded image instead.

### Total Kidney Volume Computation

All CT data sets were manually segmented by clinical experts and trained personnel in order to obtain ground truth annotations of the kidneys. Before computing TKV from individual segmentations obtained from the CNN, we first resampled the 224 × 224 segmentation predictions back to original size of 512 × 512 using bicubic interpolation method. Then, we performed a morphological closing operation to recover potential holes within predicted kidney regions and to remove any small isolated noise pixels wrongly predicted as foreground (kidney) pixels. Finally, TKVs were computed as the product of number of foreground pixels multiplied with the pixel spacing in x and y direction and the corresponding slice thickness.

### Statistical analyses

In order to evaluate the performance of our automated segmentation method, the DSC^41^ was used as a statistical validation metric to assess the spatial overlap accuracy of the predicted and true manual segmentation labels. The CCC measure was used to evaluate the reliability and reproducibility of the automated method with respect to the standard manual method. Furthermore, a BlandAltman analysis was used to assess the agreement between TKV estimated from the automated segmentation method and the corresponding manual segmentation. Both absolute and relative differences were computed on the Bland-Altman plots. The COV for repeated measures^42^ (computed as the ratio of standard deviation to mean of the measurements) between true and automated TKV was also computed. The non-parametric Wilcoxon signed rank test for paired samples was used to measure the statistical significance of correlation between automated and true TKV measurements. Additionally, Spearman’s rank correlation coefficient (*ρ*) was employed as a non-parametric test to measure the strength of association between the automated and true TKV measurements. Finally, we computed the MAPE and RMSE with respect to TKV in order to provide error measures which are not sensitive to under-estimations and over-estimations cancelling out each other. All statistical analyses were performed using R studio^43^ version 0.98.953.

## Electronic supplementary material

Supplementary Information Automatic Segmentation of Kidneys using Deep Learning for Total Kidney Volume Quantification in Autosomal Dominant Polycystic Kidney Disease
