# Supplementary material for: Automatic Segmentation of Kidneys using Deep Learning for Total Kidney Volume Quantification in Autosomal Dominant Polycystic Kidney Disease
Source: Sci Rep. 2017 May 17;7:2049. doi: 10.1038/s41598-017-01779-0 (PMC5435691; doi:10.1038/s41598-017-01779-0)
Supplement: Supplementary file 1 — Supplementary Information Automatic Segmentation of Kidneys using Deep Learning for Total Kidney Volume Quantification in Autosomal Dominant Polycystic Kidney Disease [file 41598_2017_1779_MOESM1_ESM.pdf]

Supplementary Information
Automatic Segmentation of Kidneys using Deep Learning for Total Kidney Volume Quantification in Autosomal Dominant Polycystic Kidney Disease

Kanishka Sharma[1,2,*], Christian Rupprecht[2,5], Anna Caroli[1], Maria Carolina Aparicio[1], Andrea Remuzzi[1,3], Maximilian Baust[2], and Nassir Navab[2,4]

[1]Clinical Research Center for Rare Diseases "Aldo e Cele Daccò",
IRCCS-Istituto di Ricerche Farmacologiche "Mario Negri", Ranica, 24020, Italy
[2]Computer Aided Medical Procedures, Technische Universität München, Garching, 85748, Germany
[3]University of Bergamo, Bergamo, 24129, Italy
[4]Computer Aided Medical Procedures, Johns Hopkins University, Baltimore, MD 21218, USA
[5]Department of Computer Science, Johns Hopkins University, Baltimore, MD 21218, USA
[*]kanishkasharma10@gmail.com

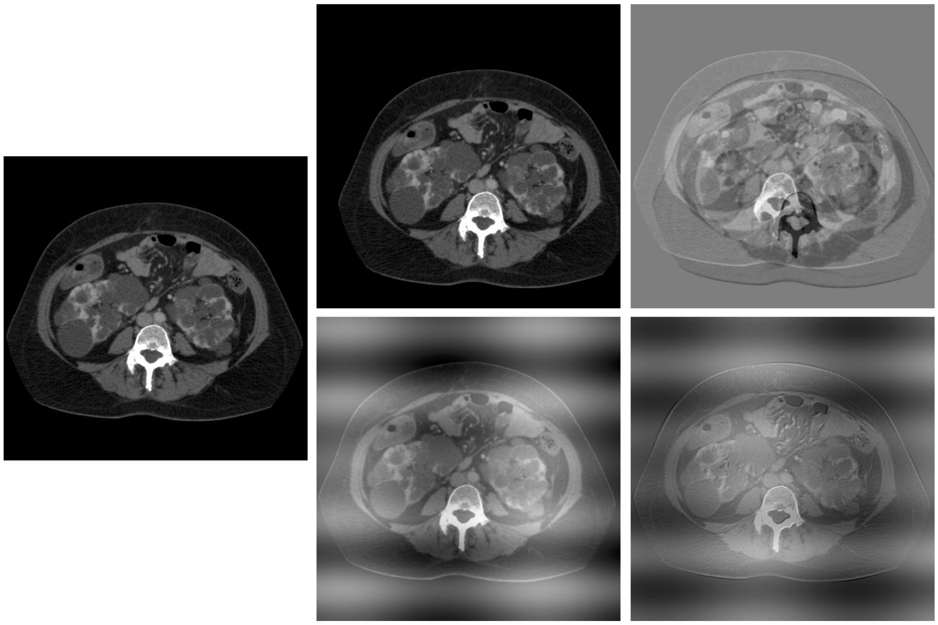

Figure S1. Data Augmentation: Left: Original patient CT image; Top Centre: Image obtained by first augmentation strategy, Top Right: Difference image from orginal and shifted image; Bottom Centre: Image obtained by second augmentation strategy, Bottom Right: Difference Image from original and transformed (deformation) image.

Data Augmentation

The data augmentation step was performed to mitigate overfitting and to achieve a good generalisation by using two independent stategies for data augmentation. In the first type of augmentation, the shift image was generated by rigid translation of 32 pixels each in x and y direction (Figure S1 (Top centre)).
In the second type of augmentation, we applied mild non-rigid deformations to the input image and added low frequent intensity variation to obtain the final augmentation (Figure S1 (bottom centre)). Both augmentation methods were performed using commercial software package Matlab[1]. The functions below describe the second strategy for altering input image I.

Generate deformation field on the original input image
```
[x,y] = meshgrid(linspace(0,2*pi,size(I,2)),linspace(0,2*pi,size(I,1)))
D = cos(a*rand(1)*x) + sin(a*rand(1)*x) + cos(a*rand(1)*y) + sin(a*rand(1)*y)
D(:,:,2) = cos(a*rand(1)*x) + sin(a*rand(1)*x) + cos(a*rand(1)*y) + sin(a*rand(1)*y)
```

Warp Image
```
Iw = imwarp(I,D,'linear','FillValues',0)
```

Add low frequent intensity variation
```
Iv = 0.15*(cos(a*rand(1)*x) + sin(a*rand(1)*x) + cos(a*rand(1)*y) + sin(a*rand(1)*y))
It = Iw + Iv
It = It/max(It(:))
```

Threshold Selection

In order to shed some light on threshold selection we computed the Accuracy, Precision, F1 Score and the Youden-Index (to maximize both sensitivity and specificity). Our results indicate that 0.5 yields the best compromise of the metrics and has therefore been selected for generating the final segmentation results. The results from the analysis on different thresholds have been summarised in Supplementary Figure S2.

**Threshold**	Sensitivity	Specificity	Youden Index	Accuracy	Precision	F1 Score
0.1	0.97	0.95	0.92	0.95	0.60	0.72
0.2	0.95	0.98	0.93	0.98	0.75	0.83
0.3	0.93	0.98	0.92	0.98	0.80	0.85
0.4	0.92	0.99	0.90	0.98	0.83	0.86
0.5	**0.90**	**0.99**	**0.89**	**0.98**	**0.85**	**0.87**
0.6	0.88	0.99	0.87	0.98	0.87	0.87
0.7	0.86	0.99	0.85	0.98	0.89	0.87
0.8	0.82	1.00	0.82	0.98	0.91	0.86
0.9	0.71	1.00	0.71	0.97	0.91	0.79

Figure S2. Threshold Selection: Qualitative metrics for different thresholds. As shown in the figure, 0.5 provides the optimal cut-off for threshold selection.

References

1. MATLAB. *version 8.6.0 (R2015b)* (The MathWorks Inc., Natick, Massachusetts, 2015).
